# Healthcare worker burnout: exploring the experiences of doctors working in a maternity unit in Namibia

**DOI:** 10.1186/s12913-024-10845-z

**Published:** 2024-03-21

**Authors:** Tanya Y. Brückner, S. Heemelaar, T. Endjala, T. van den Akker

**Affiliations:** 1 Athena Institute, Vrije Universiteit, Amsterdam, Netherlands; 2 Department of Obstetrics and Gynaecology, Leiden University Medical Centre, Leiden, Netherlands; 3 Department of Community and Mental Health Nursing, University of Namibia, Windhoek, Namibia

**Keywords:** Burnout, Healthcare worker, Blame culture, Support, Trauma, Maternal health

## Abstract

**Background:**

Globally, healthcare workers (HCWs) in maternity units are at high risk of developing burnout. Burnout can lead to multiple harmful impacts on HCWs, their patients, and the broader healthcare system. Little is known about the burden of burnout among sub-Saharan African HCWs. Although evidence suggests that maternity unit doctors in a hospital complex in Namibia are at risk of developing burnout, no studies have been conducted on doctors in this department yet.

**Methods:**

Through participant observation and a mixed-methods needs assessment, this study aimed to explore the drivers, experiences, and impact of burnout symptoms among doctors in this department, and current support mechanisms in place. Survey data was collected from 18 participants and seven in-depth interviews were conducted. Burnout risk was assessed using the Burnout Assessment Tool.

**Results:**

Seven out of 18 participants were at very high risk for burnout and three were at risk, showing a high prevalence of burnout risk. Burnout risk remained similar between levels of staff, while gender qualitatively impacted burnout-related experiences. Drivers of burnout were identified at personal, occupational, and systemic levels.

**Conclusions:**

Over half of participants were at risk or at very high risk of burnout. Results highlighted a need for support and identified areas for intervention and further research. Such areas include blame culture, lack of trust between colleagues, and systemic drivers of burnout. This study contributes to the understanding of burnout among HCWs in sub-Saharan Africa.

**Supplementary Information:**

The online version contains supplementary material available at 10.1186/s12913-024-10845-z.

## Introduction

Globally, work-related stress among healthcare workers (HCWs) is a major concern and increased due to effects of the COVID-19 pandemic [1–3]. If not successfully managed, it can lead to burnout, as can long-term exposure to emotionally demanding work [4]. Burnout is associated with decreased emotional and physical wellbeing, such as Post-Traumatic Stress Disorder (PTSD), and may contribute to decreased quality of care, reduced patient satisfaction, and poorer rapport between staff [5, 6]. HCWs are especially at risk of developing burnout due to factors like performing emotionally taxing work in a pressurised environment, compounded by high workloads and low social support [7].

Among different specialty disciplines, maternity care workers (MCWs) experience some of the highest levels of burnout [8]. MCWs have an especially high exposure to traumatic events like the loss of a baby or woman. Exposure to such traumatic events can increase levels of burnout [9, 10]. The high prevalence of burnout in maternity units is likely exacerbated by working in a high-risk context, delivering more acute care, and working heavy night shifts [4].

While burnout is prevalent among HCWs across the globe, little is known about the burden of burnout among HCWs in sub-Saharan African (SSA) [4]. Namibia is an upper-middle-income country in SSA with a maternal mortality ratio estimated at 215 per 100,000 live births in 2020, compared to the upper-middle-income country average of 61 per 100,000 [11, 12]. A shortage of qualified HCWs, compounded by high staff turnover and limited staff experience and supervision, is an important contributor to maternal mortality in Namibia [13, 14]. High workload and stress among Namibian HCWs were previously found to be associated with the provision of disrespectful maternal care [15].

There is a high prevalence of exposure to adverse events in two of the largest hospitals in Namibia, which form one hospital complex. These hospitals have the highest recorded numbers of maternal and perinatal deaths in the country [16]. Considering these facts in light of existing knowledge on contributors to HCW burnout, maternity care doctors in the hospital complex are at risk of developing burnout and experiencing its associated effects. A recent study conducted in Namibia [17] suggests high rates of burnout-related experiences among midwives. However, to our knowledge, no studies have been conducted on burnout among Namibian doctors to date.

Dyrbye and colleagues’ synthesised Three-Part Model of Physician Burnout was used as a conceptual framework to better situate this study in the broader knowledge context. We sought to investigate the key domains of personal, local and systems drivers of burnout [18]. The framework recognises the intersection of multi-level and intersectional factors that are both within and outside of an individual’s control to change. While individual factors do play a role, organisational and systemic factors are key drivers in the development of burnout [19]. Thus, this study pays some attention to personal factors like coping mechanisms, but places more emphasis on organisational and systemic factors, like available support and interpersonal work relationships.

This research aimed to explore the drivers, experiences, and impact of burnout symptoms among doctors in maternity units at the hospital complex. To better understand their needs, we also sought to explore the current support mechanisms in place in the department, as well as how doctors cope with work-related stress. Through performing a context-specific needs assessment, findings from this study could form the basis of much-needed contextually-applied interventions or other efforts to counteract burnout and optimise HCW wellbeing.

## Methodology

### Research design

This study used methodological triangulation by including participant observation with a concurrent mixed-methods design consisting of a survey to assess the prevalence of burnout symptoms and in-depth interviews to explore doctors’ experiences of working in the maternity unit. Survey and interview data were collected concurrently as it allows for each method to strengthen the other, thereby deepening the researchers’ understanding of the findings as well as providing greater validity [20]. Data were collected in February and March 2021 by the primary researcher, who is Namibian. Data collection involved all relevant stakeholders and was conducted in English.

### Study setting

The hospital complex consists of the only tertiary care centre in the country, as well as a regional hospital. Doctors working in the obstetrics and gynaecology department are responsible for the obstetric and gynaecological care in both hospitals. Both hospitals have independent gynaecology wards as well as maternity units. During the day, doctors are divided into teams and provide either obstetric or gynaecological care in one of the two hospitals. When on call, doctors cover one hospital for obstetrics and gynaecology, but still sometimes have to travel between the hospitals, as the blood bank or intensive care, for example, is only available in one hospital, or because there are not enough doctors to cover each hospital.

The number of doctors in the department fluctuates depending on availability of funds from the Ministry of Health and Social Services to fill vacancies. Hospitals often do not have enough funds to hire replacements for those on study leave or to create more vacancies. While the average working hours per week are 45, as defined by the Labour Act 11 of 2007, HCWs are regarded as essential workers and are allowed to surpass this. The Labour Act does not stipulate a limit on the number of hours one can work overtime as an essential worker.

### Sample population information

Total population sampling was used in this study. Six hierarchical positional groups were present among the doctors working in the obstetrics and gynaecology department: the Head of Department (HoD), specialists in obstetrics and gynaecology, Senior Medical Officers (SMOs), Medical Officers (MOs), Medical Intern team leader(s), and Medical Interns. For the sake of anonymity, stakeholders were clustered into three groups: specialists, MOs, and interns. Representatives from each group were included in the study.

When complications arise, interns are called to assess the patient first. If they need further help, they should contact the MO on call. The department has 14 to 18 MOs on average. One of the MOs acts as an intern coordinator, who works more closely with the interns than the SMOs do. The HoD is one of the specialists. At the time of study, there were 54 doctors working in the department: nine specialists, 18 MOs, and 27 interns.

### Participant observation

The primary researcher, who was part of the participant observers, identified and oriented herself to the current department processes and dynamics. She also met with key stakeholders, who had been pre-identified prior to commencement of the study. These stakeholders introduced the primary researcher to other doctors in the department. During this phase, she identified and purposefully recruited key stakeholders for interviews and identified relevant stakeholder groups to include in the survey. Key stakeholders were identified according to position in the department.

### Needs assessment

After observations, the self-administered survey was disseminated among all relevant stakeholders in the department. Initially, an online survey link was disseminated via a key stakeholder on WhatsApp groups, but this received few responses. To increase the response rate, physical copies with a secure box for collection were placed in the conference room where daily handover meetings took place. One interview participant was recruited through the survey through the use of an anonymous link.

The survey consisted of seven parts. It was previously developed and used in the Netherlands [21] and adapted to the Namibian context. This was done through discussion and by cross-referencing the questionnaire with that of one of the authors (T.E.), who performed a similar study on midwives in the department [17]. The survey included the Trauma Screening Questionnaire, a validated 10-item screening instrument that indicates provisional diagnosis of PTSD according to the DSM-IV [22]. Questions on burnout were adapted from the validated Burnout Assessment Tool (BAT-23), chosen due to its ability to address the conceptual, psychometric, and practical applicability limitations of the well-known Maslach Burnout Inventory tool (Schaufeli W, De Witte H, Desart S: Manual Burnout Assessment Tool (BAT) - Version 2.0, unpublished internal report). It was recently validated in South Africa [23], proving its applicability to the Namibian context. The final survey was piloted among non-participating HCWs who had previously worked in the department for face validity, after which some amendments were made.

Semi-structured interviews were also conducted with key stakeholders in-person or through video conferencing tools. The interview guide was created based on a guide developed by one of the authors (T.E.), whose questionnaire also informed the data collection tools [17]. This ensured that the guide was contextually sensitive and included relevant questions. The interview was reviewed and piloted four times prior to data collection with different non-participating HCWs who had previously worked in the department, and subsequently refined. Interviews were conducted on a voluntary basis. The time ranged between 30 and 55 min, depending on the availability of participants and on when data saturation was reached.

### Data analysis

Interview data was analysed as qualitative data, and survey data was analysed as quantitative data. The survey results were inductively coded for open-ended questions and closed-ended questions were analysed using descriptive statistics in IBM SPSS Statistics (Version 26). Groups were compared according to position, gender, and age, using measures of central tendency and frequency distribution.

PTSD prevalence was calculated based on methods used by Baas et al. [21], which calculated the Trauma Screening Questionnaire outcomes with a cut-off value of six or more [24]. The sample size was too small to test for statistical significance or associations.

The BAT-23 results were analysed according to the guidelines in the user manual (Schaufeli W, De Witte H, Desart S: Manual Burnout Assessment Tool (BAT) - Version 2.0, unpublished internal report). A global burnout score was calculated for each participant, based on the four core symptoms (exhaustion, mental distance, emotional impairment, and cognitive impairment). Scores for psychological distress and psychosomatic complaints were combined into one score labelled ‘secondary symptoms’. The average score for each dimension was calculated too, to provide a differentiated overview for more detailed insight into participant experiences. The scores were interpreted using clinical cut-off values, which compares the participants’ scores with those of patients who have been diagnosed as burned-out by trained professionals. This establishes an individual’s level of risk of burnout.

All interviews were voice-recorded and manually transcribed. Thematic analysis was conducted on ATLAS.ti 8 software using integrated inductive and deductive coding. Codes were inductively clustered into themes. Triangulation of interview results with survey results and participant observations ensured validity, as did member-checking. To assess inter-rater reliability, data analysis was discussed during academic supervisory team meetings. In these meetings, results were discussed with research team members who had previously worked in the department and had had similar experiences to the participants, and results were compared to previous research conducted by author T.E. in the same department with a different study group.

### Ethics

The study received approval from the Chief Medical Superintendent of the hospital complex. Ethical approval was received from the Research Management Committee at the Namibian Ministry of Health and Social Services (Ref: 17/3/3/TYB). Written and verbal informed consent was obtained from all participants prior to participation. Data confidentiality was ensured by proper data storage on a personal password-protected data storage website. Anonymity was ensured by excluding identifiable information from data, using pseudonyms and anonymised questionnaire links. Flexible scheduling allowed for justice in participation.

This research preceded the co-development and implementation of a staff support structure to mitigate burnout as part of the same study. In this way, additional support was provided. The primary researcher also emphasized the availability of psychosocial professionals in the hospitals, should participants require further support.

## Results

### Sample population information

Of the 54 doctors in the department, 26 agreed to participate in the survey. Eight responses were removed for being blank or incomplete. Thus, the survey received 18 valid responses. Seven participants agreed to take part in the interviews. Survey participants will be referred to as participants ‘a’ and interview participants will be referred to as participants ‘b’. Participant characteristics are presented in Table 1.

**Table 1:** Characteristics of sample population

Variables		Survey participants (a) (N)	Interview participants (b) (N)
Total sample		18	7
Gender	Male	5	3
	Female	12	4
	Non-binary	1	-
Marital status	Single	12	n/a
	Married	5	n/a
	Other	1	n/a
Position	Medical Intern	10	2
	Medical Officer	7	4
	Specialist	1	1

### Associated mental ill-health: PTSD prevalence

Of 18, 15 participants (a) reported having experienced at least one adverse event (maternal and/or perinatal death) during their work in the last six months and thereby meet the DSM-IV criterion A of PTSD diagnosis. Three screened positive for a current PTSD diagnosis. Among them, one was an intern and two were MOs. All participants who screened positive were female. Additionally, seven participants (a) reported having experienced multiple work-related PTS-symptoms earlier in their career.

### Burnout prevalence

Participants (b) often described burnout symptoms, especially the experience of exhaustion (physical and mental), irritability, and feeling mental distance from work. On average, on a Likert scale of one to five, participants (a) scored 2.68 on the burnout score (core symptoms). See Table 2. According to cut-off values, this means that participants are at risk for burnout. The mean was calculated for each symptom category and was also analysed according to cut-off values. ‘Exhaustion’ received an average score of 3.69, which is classified as ‘at very high risk’, according to cut-off values. This was followed by mental distance (x̅ = 2.6) and emotional impairment (x̅ = 2.21), which are categorised as at risk’ scores for burnout. The remaining symptom category averages, namely for cognitive impairment and secondary symptoms, were deemed as ‘not at risk’.

**Table 2:** Burnout score averages

Participant characteristics			Burnout symptoms					
Participant	Gender	Position	Total (core symptoms)	Exhaustion	Mental distance	Emotional impairment	Cognitive impairment	Secondary symptoms
P1	Female	MO	3.04^a^	3.75^a^	3.6^a^	**2.4**	2.4	2.7
P2	Female	MO	3.4^a^	4^a^	3.6^a^	3^a^	**3**	4.3^a^
P3	Male	Intern	2.58	3.5^a^	**2.8**	2	2	1.7
P4	Male	MO	3.25^a^	4.38^a^	**2.6**	3^a^	**3**	2.2
P5	Female	Intern	2.47	3.88^a^	3.6^a^	1.2	1.2	2.2
P6	Male	Specialist	1	1	1	1	1	1
P7	Female	MO	**2.87**	3.88^a^	**2.8**	**2.4**	2.4	2.1
P8	Female	Intern	3.94^a^	4.75^a^	3.4^a^	3.8^a^	3.8^a^	3.4^a^
P9	Female	Intern	3.07^a^	4.88^a^	3.8^a^	1.8	1.8	3.4^a^
P10	Female	Intern	2.04	**2.75**	1.4	2	2	2.1
P11	Female	MO	1.98	3.5^a^	1.6	1.4	1.4	1.4
P12	Female	MO	2.31	3.25^a^	2	2	2	2.1
P13	Male	MO	1.78	3.13^a^	1.2	1.4	1.4	1.6
P14	Female	Intern	2.55	4.38^a^	**2.6**	1.6	1.6	2.8
P15	Non-binary	Intern	3.02^a^	3.88^a^	2.2	3^a^	**3**	**3.2**
P16	Female	Intern	**2.91**	4.25^a^	**2.6**	**2.4**	2.4	2.8
P17	Male	Intern	3.14^a^	3.38^a^	3.2^a^	3^a^	**3**	2.4
P18	Female	Intern	**2.87**	3.88^a^	**2.8**	**2.4**	2.4	2.7
Average scores			**2.68**	3.69^a^	**2.6**	**2.21**	2.21	2.45

High-risk scores are represented in bold. Note: participant (a) codes do not correspond to participant (b) codes

The superscript ^a^ denotes a “very” high-risk score

Stratifying the burnout symptoms with demographic variables provided further insight into the prevalence of risk of burnout. According to the global score, seven out of 18 participants were at very high risk for burnout, three were at risk, and eight were not. Three of the seven high risk participants were MOs and the remaining four were interns. Of the three participants who were at risk of burnout, one was an MO and two were interns. Therefore, although the sample size is too small to run correlation analysis, results demonstrate that the divide between interns and MOs was similar.

### Burnout drivers: Personal domain

#### Experiences of adverse events

**Fig. 1:**
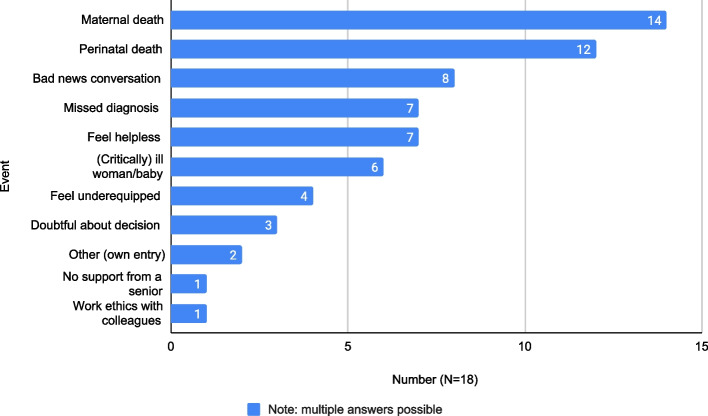
Work-related events considered the most emotional

Fourteen out of 18 participants (a) considered maternal deaths the most emotional work- related events, and 12 considered perinatal deaths the most emotional. See Fig. 1.

While participants (b) generally enjoyed their work, 10/18 participants (a) had seriously considered quitting their job in the department. Of seven participants (a) who stated why they had considered doing so, six identified the high rate of exposure to adverse events as the reason, while one participant stated that it was due to the hostile work environment. Participants (b) described adverse events as traumatic and emotionally draining, and experienced self-blame, depressive moods, and suppression of emotions. They often did not have the emotional capacity to process adverse events. A participant, still ruminating about an event that happened years earlier, described their experience several years later: ‘You have to see the next patient and when you are done […] your mind is totally dead, you just want to sleep. So, I don’t think I did much with it. Probably that’s why it keeps popping up.’ (P1).

#### Self-reported quality of care

Some participants (b) reported feeling that their quality of care was reduced due to burnout-related symptoms. They described instances where they themselves or their colleagues were so exhausted that they fell asleep in front of patients, became more irritable, missed important observations, were more prone to making mistakes, or failed to adhere to protocol: ‘That can definitely affect your patient care because you’re not there, you’re more prone to… make mistakes or overlook things, maybe cut corners if you’re very, very tired, just to get the work done. It can be very detrimental.’ (P2).

Additionally, participants stated that poor relationships between colleagues negatively impacted willingness to help each other cover shifts if they were sick or in desperate need of rest, which means that one would have to continue to provide care even when one considered oneself unfit to do so.

#### Coping

**Fig. 2:**
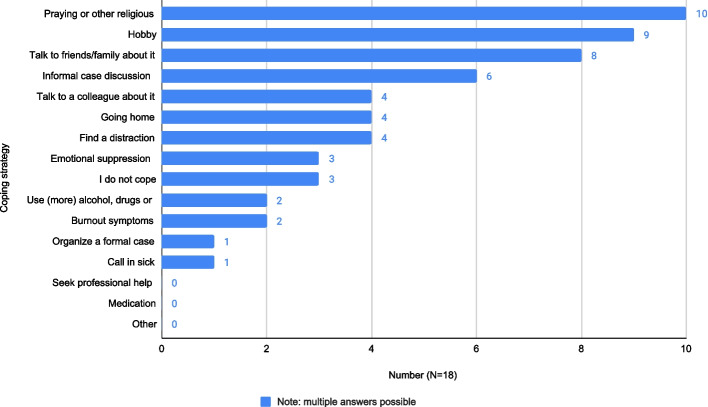
Coping strategies

The commonest ways of coping with emotional events at work were reported by participants (a) as praying (n = 10), performing a hobby (n = 9), talking to friends or family (n = 8), and having an informal case discussion (n = 6). Of 18, six participants spoke to colleagues about the event, and none sought professional help (Fig. 2).

### Burnout drivers: Local (organisational) domain

#### Blame culture

Following an adverse event, case discussions occurred soon after in the daily handover meeting or, in severe cases, in an ad hoc case review meeting. However, the way in which the discussions unfolded was often perceived by participants (b) as a ‘witch hunt’: aggressive, hostile, and asserting blame, resulting in those on the receiving end becoming defensive. Some participants acknowledged that while there is a tendency to shift towards blame when discussing cases, there is also the reality that some doctors lack skills, and those responsible must be held accountable. Nonetheless, participants (b) noted a fine line between being held accountable for one’s actions and being made to feel bad about oneself:

I know I should learn from it, [...] but if now I already feel terrible about what I did,

and then I have the whole team now sort of putting me in a corner and pointing

fingers at me, that might just push me off the edge and I become depressed. [...]

Tomorrow I might not want to come back to work. (P3)

According to a participant (b), blame culture deters others in the department from helping in an emergency case as they feared both the verbal repercussions of accusatory blame and the trauma associated with the incident:

…People don’t want to get involved because they just don’t want that trauma in their life, or they don’t want that drama or they don’t want to be involved in an incident where they have to explain what has happened, explain what was who doing and what not, because you fear the questions coming instead of fearing losing the patient. (P4)

#### Lack of trust

Participants (b), especially interns and MOs, reported feeling a lack of trust in their colleagues. This discouraged participants from speaking to peers about challenges they might be facing, as they felt others might use that information against them somehow. Participants (b) thought that improving feelings of trust and support between co-workers would be beneficial not only to interpersonal work relations, but also to their personal experiences of work:

If we can have that, it won’t be a problem coming to work, knowing it’s stressful, you’re exceeding your hours, but you’re ok with being at work, you’re happy spending an extra hour or two just to help somebody. [...] Because you know that person has your back also at work, and at any given time. (P4)

#### Institutional support

According to all participants (a and b), there is no protocol available in the department regarding support after an adverse event. Thirteen participants (a) said they received no support after a maternal or perinatal death. Additionally, eight participants (a) disagreed that the institutional support was good, nine stated that there was no support organised, and one agreed that the support was good. When asked if there should be a change in the department or institution regarding support after an adverse event, four participants (a) agreed and fourteen strongly agreed. None disagreed. Additionally, a female participant (b) described a lack of consideration and support after her short maternity leave as distressing and majorly contributing to feelings of burnout and depression.

However, participants (b) described feeling some support from specialists and the SMOs. They thought that specialists provided good academic support, which helped with ease of work in terms of knowing what to do in certain medical situations. They also thought that SMOs were highly supportive in the capacities they had available to them. For example, they provided some flexibility and choice with time off for personal reasons.

### Burnout drivers: Systems domain

#### Understaffing and workload

Participants (b) reported that short staffing significantly contributed to high stress levels. Some doctors described being expected to be in two places at the same time, which is especially problematic when complications arise that can lead to maternal deaths, perinatal deaths, and severe morbidity. In addition, high workload was frequently stated as a common reason why participants (b) have considered leaving the department or not re-joining it after their internship. Two female participants (b) were unhappy about missing spending necessary quality time with their children and families due to the workload. They described feeling overwhelmed by the double burden of (domestic and professional) care.

Working hours in the department varied by occupation level and were influenced by factors like reallocation of personnel to the COVID-19 unit, colleagues taking study or personal leave, and the availability of funds for the department from the MoHSS. At the time of this study, participants worked between five and seven days a week, and were on call six or seven times per month on average. Day shifts were officially nine hours long each, but participants (b) described often working for longer. On calls lasted over 24 h. One participant (b) described working over 80 h some weeks.

## Discussion

To the best of our knowledge, this is the first study on burnout among maternity care doctors in Namibia. It is also the first known study using the Burnout Assessment Tool to measure burnout among SSA HCWs. Findings highlighted a high prevalence of burnout-related experiences among this population, where more than half were at risk or at very high-risk of burnout. While the small survey sample size limited statistical analysis possibilities, descriptive and interview data provided valuable insight into experiences of burnout-related symptoms according to gender and occupational position as well as prevalence of individual symptom categories.

Results highlighted the potential influencing impact of gender on burnout, pointing to wider systemic contributors to burnout. Conducting further research on this difference would be valuable in contributing a Namibian perspective to literature about the gendered experiences of burnout among female maternity care doctors, where women have reported higher levels of burnout than men, especially related to emotional exhaustion [25, 26]. For example, female MCWs in Kenya have reported higher burnout scores compared to their male counterparts [27]. Findings support global discussions of the double burden of care: not only are female MCWs often expected to perform more child- and domestic-care, but they are also likely to experience bias and discrimination related to pregnancy or maternity leave and a lack of support both during pregnancy and after childbirth [28].

Results also presented insights into individual burnout symptom categories. Similarly to other studies conducted on HCWs in SSA [29], participants frequently described feeling mentally distant from their work, mentally and physically exhausted, and experienced psychological complaints such as feeling anxious. However, it is interesting to note that almost all participants had a very high-risk score for exhaustion, despite almost half not meeting the global score for being at-risk or at very high-risk of burnout. High exposure to traumatic events with inadequate subsequent institutional support can lead to high rates of emotional exhaustion [30]. Additionally, the theory of allostasis states that chronic exposure to traumatic experiences and stressful events can lead to exhaustion [31].

Differences in development of burnout may be attributed to individual diversity in internal resources. Those with fewer internal resources may be more susceptible to the effects of chronic stress and high trauma exposure as they are less able to cope with the effects thereof [32, 33], though cultural differences may also play a role. In cultures that practise high religiosity, there may be reduced allostatic load due to regular attendance of religious services [34]. In this study, praying was the commonest coping mechanism.

Nonetheless, results highlighted the impact of high exposure to occupational stressors like deaths, blame, and a lack of trust. Almost all participants were involved in the care of at least one woman and one baby that died around birth over the past six months. These were considered the most emotional work-related events and described as traumatic and leading to feelings of negativity and self-blame. Three participants screened positive for a PTSD diagnosis, while several others experienced numerous work-related PTS-symptoms earlier in their career. Literature shows that burnout is associated with decreased wellbeing such as PTSD and may contribute to decreased quality of care and high turnover rates [5, 6]. Midwives in the department reported similar experiences; some wanted to leave the profession due to the high exposure to adverse events, and many experienced self-blame and guilt. Their family lives were negatively affected, and they experienced increased feelings of loneliness, which can lead to further distress and depression [17].

Despite the high exposure to stressors, participants described receiving no support after an adverse event from the department. Such experiences increase the risk of burnout, as factors like performing emotionally taxing work in a pressurised environment are compounded by high workloads and low social support [7]. For example, low supervisor support contributed to higher burnout levels among East African nurses [35]. Not only so, but participants also received blame for such events, which contributed considerably to negative affect. Midwives in the same department reported similar experiences of the blame culture, including being blamed and stigmatised by the general public after a death event [17].

Interpersonal aggression like blame culture can trigger a stress response and feelings of anxiety, depression, job dissatisfaction and burnout [36]. This response is positively associated with depersonalization (mental distance) and emotional exhaustion, both of which were frequently experienced by participants. Similarly, interpersonal conflict at work was found to be a predictor of burnout among nurses in South Africa [37]. Findings also showed that participants feared being blamed more than the act of making an error. Fearing being blamed can ultimately impose a barrier to reporting errors, thus limiting opportunities for improvement, and resulting in high turnover [38].

The causes of blame culture are likely multifaceted and common in many countries globally [39–41]. Individuals are held responsible for errors, as opposed to the systems in which they work. A key component of medical culture is medicine’s standards of perfection and invulnerability [42], leading individuals in this system to be more vulnerable to the impact of a blame culture like loss of professional reputation [43]. However, it is likely that burnout and its drivers in this setting affected the continuation of a blame culture. All levels of staff throughout the hospital complex face similar systemic drivers of burnout: despite servicing over 80 percent of the Namibian population, the public healthcare sector faces major resource deficiencies [44, 45]. Considering that, as managerial figures, senior staff are held accountable for those working under their supervision, it is likely that they transfer blame to those who were directly involved in an effort to avoid the harsh blame on themselves.

The transference of blame is not a unique phenomenon to Namibia nor to the healthcare context: a common organizational strategy used to manage blame is to create scapegoat(s) to avoid blame and its associated repercussions [46]. In Namibia, HCWs have faced public scrutiny and legal procedures, exacerbating the fear of blame. Media reports have gone so far as to directly accuse individual HCWs of the death of a woman or baby in national newspapers [47–52]. Understanding the systemic contributors to human error, such as exhaustion from working in high-stress and low-resource settings, is essential in distinguishing between inevitable human error and blameworthy behaviour [53]. To contribute to addressing the fear of blame among HCWs, the national maternal and perinatal mortality committee of Namibia focused on the identification of system failures, rather than on mistakes made by individuals [47].

### Strengths and limitations

This study had a small sample size, which did not allow for quantitative data analysis showing causal relationships and statistical significance. Underreporting may have occurred due to the possibility that the nature of explored experiences was considered unfavourable, undesirable, or stigmatised. Similarly, interview participants may have provided socially desirable answers for the same reasons. Responder bias may have occurred as people who thought they experienced burnout-related symptoms may have been more likely to fill out the survey than those who did not think so. Additionally, the results are not representative of maternity care doctors in other centres in the country, thus limiting generalizability. Nonetheless, findings align with both international and regional research, strengthening validity.

Strengths include using a variety of methods to answer the research question. This enabled triangulation of data as results from each method informed each other. Triangulation was also made possible through the inclusion of a number of stakeholders from a variety of occupational positions, allowing a more in-depth exploration of experiences across positions. However, only one specialist was included, disallowing meaningful comparison between this position and others.

## Conclusion

This study not only demonstrates well-known contributors to HCW burnout among Namibian doctors in the hospital complex maternity units, but also a lesser-established yet highly impactful driver: blame culture. Significant changes must be made to address blame culture to establish a sense of social safety and support. Common contributors to occupational stress included high levels of exposure to adverse incidents, a lack of trust between colleagues, and a lack of support structures. Over half of the participants were either at risk or at very high risk of burnout, pointing to an acute need to address burnout drivers in this population. Some qualitative contributors to burnout according to gender were noted, while burnout risk remained similar between MOs and interns. It is highly recommended that further studies explore how to increase feelings of support and trust between colleagues to mitigate the development of burnout symptoms.

One cannot ignore the systemic drivers of burnout. Efforts to mitigate burnout contributors cannot only be individually- or community-based. To truly make a significant difference, changes on a system-level must be included, too. Policy adjustments should be made, such as allocating more resources to public maternal healthcare to combat burnout drivers like under-staffing. The complexity of this challenge is considerable but increasing the level and distribution of resources in an under-resourced public hospital is needed. This study is a call-to-action to pay meaningful attention to and address the experiences of maternity care doctors. Not only would this improve their health and wellbeing, but it could also likely have ripple effects on their patients and, ultimately, on the wider health system in general.

### Supplementary Information

**Supplementary Material 1.**

**Supplementary Material 2.**

## Data Availability

The dataset supporting the conclusions of this article is available on request from the corresponding author. The data are not publicly available to protect the privacy of research participants.

## Abbreviations

HCWs: Healthcare workers
PTSD: Post-Traumatic Stress Disorder
SSA: Sub-Saharan Africa
SMO: Senior Medical Officer
MO: Medical Officer
HoD: Head of Department
