# Supplementary material for: Healthcare worker burnout: exploring the experiences of doctors working in a maternity unit in Namibia
Source: BMC Health Serv Res. 2024 Mar 21;24:362. doi: 10.1186/s12913-024-10845-z (PMC10958874; doi:10.1186/s12913-024-10845-z)
Supplement: Supplementary file 1 — Supplementary Material 1. [file 12913_2024_10845_MOESM1_ESM.docx]

**Interview guide**

Good morning/afternoon! Thank you for agreeing to this interview.

I’m doing this project so I can gain a better understanding of the way the hospital support processes work, gain insight into staff experiences with burnout and overall coping with stressful situations, and develop possible solutions and ways to improve a sense of support and coping. So your participation in this interview is really helpful to me, thank you for agreeing to it! I’m very interested in what you have to say.

This interview will take approximately 45-55 minutes and we’ll start with some basic questions about yourself, some questions about the departmental processes, your experience with burnout and coping, and possible ways forward.

I would like to make sure that you know that your identity will be protected and anonymous and you don’t have to answer any questions you don’t feel comfortable to. You can also decide to stop the interview at any stage if you feel uncomfortable.

Do you have any questions for me before we begin?

Is it ok with you if we record this interview?

**Section 1: Background information**

1. To begin, could you briefly introduce yourself please?

- *Prompts if not brought up by participant:*
  - How old are you?
  - What is your current position at the hospital?

1. How long have you been working for in Obs&Gyn?
   1. Do you enjoy it?

**Section 2: Hospital/departmental processes**

I’d like to find out some information about how the department works and how you experience this.

1. What does your normal working day look like?
2. How many ward hours do you work a week/month?
3. How many on call hours do you work a week/month?
4. Are there teams that work together in the department?
   1. If yes, how are the teams formed? (who is in each team) (is this a mix of e.g. interns, MOs, nurses?)
5. What do debriefings after an emergency or adverse event (e.g. maternal death, near death) entail?
   1. *Prompt*: What happens?
   2. (How do you experience these?) – do you think they are helpful, is there anything else you would like from these?
6. Is there any kind of support organized by the department or hospital after an emergency situation or adverse event?
   1. What does it look like? (what does it entail)
   2. How do you reach this support?
   3. If yes: Have you utilized this support?
      1. Prompt: why/why not?
   4. If yes: How do you perceive this support?
      1. *Prompt:* Is it good, bad, could be better…?

**Section 3: Burnout and mental health**

I’m going to ask some questions now about your experience with burnout and general coping. If you feel uncomfortable answering any of these questions, you don’t need to answer them - please just tell me if you’d prefer not to answer them. I’m going to proceed now, is that ok?

1. How would you describe your current feelings towards work?
   1. Do you feel like you are burnt out or have previously experienced burnout working in this department?
2. [Coping] Could you describe a time in which you felt any of the following regarding your job: emotionally exhausted, negative or cynical attitudes towards work, or feelings of failure?
   1. How did you cope with these experiences?
3. [Environment] What are your feelings towards the Ob/Gyn department environment? What aspects of your work environment helped you (cope with tiredness or general coping) or held you back?
4. [Resources] Are there any resources available to help with any psychological struggles you may experience?
   1. If so, are they widely used?
5. [Management] What are your perceptions regarding the relationship between management and staff in the department?
6. [Workload] Earlier you spoke about how often and for how long you work. What are your thoughts on the workload you and your coworkers experience?
   1. Prompt: is it manageable for you?
      1. Prompt: I can imagine there was a lot of sacrifice for you, would you mind telling me a bit more?
   2. Do you think it affects your quality of care?
      1. If yes, how?
7. [Typicality] Are your experiences common for someone in your role?
   1. If no: Why do you think so?
8. [Support] What are your feelings regarding support from your coworkers?
   1. In what way do you support each other?
   2. Do you talk about issues that come up at work?
   3. Is this something you’d like to have more often?
   4. How would you describe feelings of trust between colleagues?

**Section 4: Possible solutions**

Thank you for your insight so far, it’s been really valuable. I have a few last questions for you about improving the support you experience.

1. What are your thoughts about how well you cope with stress and adverse events (e.g. complication during birth) at work?
   1. Is this (your coping) something you would like to improve?
      1. Follow up: is there anything you would like to learn to do?
2. What would you need to feel more supported at work?
   1. Prompt: how could current support be improved?

Those are all the questions I have for you today. Is there anything else related to your experience that you feel you would like to share with me?

Thank you so much for participating in this interview, it has been really interesting, and I really appreciate your time and energy. If you have any questions for me after this interview, please don’t hesitate to contact me via my email address: [email address] *(have it written down for them).*
