# Supplementary material for: Healthcare worker burnout: exploring the experiences of doctors working in a maternity unit in Namibia
Source: BMC Health Serv Res. 2024 Mar 21;24:362. doi: 10.1186/s12913-024-10845-z (PMC10958874; doi:10.1186/s12913-024-10845-z)
Supplement: Supplementary file 2 — Supplementary Material 2. [file 12913_2024_10845_MOESM2_ESM.docx]

Burnout and support

Welcome to this questionnaire! You are invited to participate in a research project aiming to understand the extent and experience of burnout and its associated effects, to understand the current level of perceived support and coping, and ultimately to develop a support structure to help improve these experiences.

Burnout is a common phenomenon experienced by workers around the world and is particularly commonly seen in stressful work environments such as overburdened medical facilities. It is characterised by lack of energy, reduced professional efficacy, and negative feelings and cynicism towards or mental distance from one's job.

To begin addressing this issue, one first needs to understand the extent and the experiences of it. This research project is conducted by Tanya Brückner under the supervision of Stephanie Heemelaar and Thomas van den Akker at the Vrije Universiteit Amsterdam. Your participation in this questionnaire is voluntary and you may choose not to participate. If you decide to participate in this questionnaire, you can choose to withdraw at any time. We ensure that the questionnaire responses are completely anonymous; none of your answers can be traced back to you and no records will be kept that can personally identify you. It will take you approximately 15 minutes to complete the questionnaire. 
Please note: by completing the questionnaire, you consent to participating in it.

Section 1A: Demographics

What is your sex?

- Male (1)
- Female (2)
- Non-binary / third gender (3)
- Prefer not to say (4)

What is your age?

- 20-29 years (2)
- 30-39 years (3)
- 40-49 years (4)
- 50-59 years (5)
- ≥ 60 years (6)

What is your marital status?

- Single (never married) (1)
- Live-in partner (cohabiting) (2)
- Married (3)
- Separated (4)
- Divorced (5)
- Widowed (6)
- Other (7)

What is your current position?

- Medical intern (1)
- Medical officer (2)
- Nurse midwife (3)
- Student nurse (5)
- Midwife (4)
- Consultant gynaecologist (6)
- Other (please specify): (7) ________________________________________________

Have you worked in the department of Obstetrics/Gynecology in the last 6 months?

- Yes (1)
- No (2)

How long have you (previously or currently) worked for in the department of Obstetrics/Gynecology?

- Less than 6 months (1)
- 6-11 months (2)
- 1-2 years (3)
- 3-4 years (4)
- 5-6 years (5)
- ≥ 7 years (6)

Start of Block: Section 1B: General coping

I have seriously considered quitting my job in the department of Obstetrics/Gynecology

- Strongly disagree (1)
- Disagree (2)
- Agree (3)
- Strongly agree (4)
- I have never considered quitting my job in the department of Obstetrics/Gynaecology (5)

If applicable, what was the most important reason to consider quitting?

- I never considered quitting. (2)
- High workload (4)
- High rate of exposure to medical incidents/events (5)
- Court cases against me (6)
- Other (please specify): (1) ________________________________________________

Did you receive support from your seniors during/after this event?

- Yes (1)
- No (2)
- Not applicable (4)

What work-related events do you consider the most emotional? *(Multiple answers possible)*

- Bad news conversation/consultation (2)
- (Critically) ill woman/baby (3)
- When a woman dies (4)
- When a baby dies (9)
- When I miss a diagnosis (5)
- When I feel I can't help a woman/baby (6)
- When I'm in doubt about whether I'm making the right decision (7)
- When I feel I have to make a decision or perform procedures above my level of competency (10)
- Other (please specify) (8) ________________________________________________
- Not applicable (1)

Q20 How do you cope with the most emotional events at work? *(Multiple answers possible)*

- Seeking professional help (e.g. counselling) (2)
- Going home as soon as possible (3)
- Using (more) alcohol, drugs or cigarettes (4)
- Using new medication (5)
- Finding a distraction (6)
- Praying or other religious activities (7)
- Organising a formal case discussion with colleagues (complication meeting, perinatal audit) (8)
- Informal case discussion (discussing the case with a colleague) (15)
- Enjoying a hobby (e.g. going for a run, watching series) (9)
- Developing burn-out symptoms (e.g. emotional exhaustion, depersonalisation, less confidence) (10)
- Calling in sick (11)
- Talking to a colleague about it (12)
- Talking to a friend or family member about it (14)
- Emotional suppression (16)
- Other (please specify) (13) ________________________________________________
- I do not cope (17)
- Not applicable (1)

The current support organised by my institution after an adverse event is good.

- Strongly disagree (1)
- Disagree (2)
- Agree (3)
- Strongly agree (4)
- There is no support organised (5)

There is plenty of room to informally discuss adverse events in the department

- Strongly disagree (1)
- Disagree (2)
- Agree (3)
- Strongly agree (4)

Where did you learn to cope with adverse events? *(Multiple answers possible)*

- Medical school/nursing school (1)
- Specialist-training (2)
- By experience since beginning work in the ObGyn department (3)
- By formal training since beginning work in the ObGyn department (8)
- Other (please specify) (5) ________________________________________________
- I have never learned to cope (6)

In the course of my career, I've become more defensive about my practices (avoid high risk clinical conditions) e.g. no vaginal breech deliveries

- Strongly disagree (1)
- Somewhat disagree (2)
- Neither agree nor disagree (3)
- Somewhat agree (4)
- Strongly agree (5)

There is a protocol available at my department regarding support after an adverse event.

- No (1)
- I don't know (2)
- Yes, it consists of: (3) ________________________________________________

I have used this support before.

- Yes. (1)
- No (please explain why): (2) ________________________________________________

My preferred support after an adverse event would be: *(multiple answers possible)*

- Evaluation with the present team (1)
- Intervision (mutual group discussion) with indirect colleagues (e.g. physicians but no gynaecologists) (2)
- One-on-one conversation with a psychologist or counsellor (3)
- A buddy system/peer support (4)
- Other (please specify) (5) ________________________________________________

There should be a change of culture in my department/institution regarding support after an adverse event.

- Strongly disagree (1)
- Disagree (2)
- Agree (3)
- Strongly agree (4)

Start of Block: Section 1C: Exposure to traumatic incidents

This section contains questions about witnessing or exposure to traumatic incidents.

How many maternal deaths have you witnessed in the last 6 months that you were involved in?

________________________________________________________________

How many perinatal deaths have you witnessed in the last 6 months that you were involved in? (Fresh stillbirths or early neonatal)

________________________________________________________________

Start of Block: Section 1D: Support received

Did you receive any of the following support after the incident(s)? (multiple answers are possible)

- No support received (1)
- Debriefing session with the whole team involved (2)
- Clinical supervision (one-on-one talk with the supervisor about the incident) (3)
- Counselling from a professional counsellor (4)
- Peer support from colleagues (5)
- Other (please specify) (6) ________________________________________________
- Not applicable (7)

What kind of support did you receive from your peers?

________________________________________________________________

I am satisfied with the support I received.

- Very satisfied (1)
- Somewhat satisfied (2)
- Neutral (3)
- Somewhat dissatisfied (4)
- Very dissatisfied (5)

What is the main reason for this level of satisfaction?

________________________________________________________________

Start of Block: Section 1E: Reactions to traumatic events

Q28 Read below about reactions that can develop after a traumatic/adverse event. These questions are about your personal reactions to the event. Answer yes/no if you have experienced the following **at least twice in the past week:**

Upsetting thoughts or memories about the event have come into my mind against my will.

- Yes (1)
- No (2)
- I prefer not to answer (3)

Upsetting dreams about the event

- Yes (1)
- No (2)
- I prefer not to answer (3)

Acting or feeling as though the event were happening again

- Yes (1)
- No (2)
- I prefer not to answer (3)

Feeling upset by reminders of the event

- Yes (1)
- No (2)
- I prefer not to answer (3)

Bodily reactions when reminded of the event (e.g. fast heartbeat, stomach churning, sweatiness, dizziness)

- Yes (1)
- No (2)
- I prefer not to answer (3)

Difficulty falling or staying asleep

- Yes (1)
- No (2)
- I prefer not to answer (3)

Irritability or outbursts of anger

- Yes (1)
- No (2)
- I prefer not to answer (3)

Difficulty concentrating

- Yes (1)
- No (2)
- I prefer not to answer (3)

Heightened awareness of potential dangers to myself and others

- Yes (1)
- No (2)
- I prefer not to answer (3)

Being jumpy or startled at something unexpected that happens

- Yes (1)
- No (2)
- I prefer not to answer (3)

If you have not experienced the reactions described above recently, do you recognise them from a previous time of your life after experiencing a work-related event?

- No, I don't recognise the reactions (1)
- Yes, I do recognise having at least 6 of the reactions from a previous period in my life (2)

Start of Block: Section 1F: Depression/anxiety

The aim of this section is to find out how you currently feel. Please read every question carefully and check the box that applies the best to how you've felt for the previous week. 


Don't think about it for too long, go with your immediate reaction. There are no wrong answers - every answer is correct if it fits your own impression.

I feel tense or wound up.

- Always (4)
- Most of the time (5)
- About half the time (6)
- Sometimes (7)
- Never (8)

I still enjoy the things I used to enjoy.

- Always (1)
- Most of the time (2)
- About half the time (3)
- Sometimes (4)
- Never (6)

I get a kind of frightened feeling as if something awful is about to happen.

- Always (1)
- Most of the time (2)
- About half the time (3)
- Sometimes (4)
- Never (5)

I can laugh and see the funny side of things.

- As much as I always could (1)
- Not as much as I used to (2)
- Not at all (3)

Worrying thoughts go through my mind.

- Always (4)
- Most of the time (5)
- About half the time (6)
- Sometimes (7)
- Never (8)

I feel cheerful.

- Always (4)
- Most of the time (5)
- About half the time (6)
- Sometimes (7)
- Never (8)

I can sit at ease and feel relaxed.

- Always (4)
- Most of the time (5)
- About half the time (6)
- Sometimes (7)
- Never (8)

I feel as if I am slowed down.

- Always (4)
- Most of the time (5)
- About half the time (6)
- Sometimes (7)
- Never (8)

I get a frightened feeling like 'butterflies' in my stomach.

- Always (1)
- Most of the time (2)
- About half the time (3)
- Sometimes (4)
- Never (5)

I have lost interest in my appearance.

- Definitely (1)
- I don't take as much care as I feel I should (2)
- I take just as much care as I normally do (3)

I feel restless

- Always (1)
- Most of the time (2)
- About half the time (3)
- Sometimes (4)
- Never (5)

I look forward with enjoyment to things

- As much as I ever did (1)
- A little less than I used to (2)
- Much less than I used to (3)
- Not at all (4)

I get sudden feelings of panic

- Always (1)
- Most of the time (2)
- About half the time (3)
- Sometimes (4)
- Never (5)

Start of Block: Section 2: Core burnout symptoms

The following statements are related to your work situation and how you experience this. Please state how often each statement applies to you.

Exhaustion

|  | Never (1) | Rarely (2) | Sometimes (3) | Often (4) | Always (5) |
| --- | --- | --- | --- | --- | --- |
| 1. At work, I feel mentally exhausted. (1) |  |  |  |  |  |
| 2. Everything I do at work requires a great deal of effort. (2) |  |  |  |  |  |
| 3. After a day at work, I find it hard to recover my energy. (3) |  |  |  |  |  |
| 4. At work, I feel physically exhausted. (4) |  |  |  |  |  |
| 5. When I get up in the morning, I lack the energy to start a new day at work. (5) |  |  |  |  |  |
| 6. I want to be active at work, but somehow, I am unable to manage. (6) |  |  |  |  |  |
| 7. When I exert myself at work, I quickly get tired. (7) |  |  |  |  |  |
| 8. At the end of my working day, I feel mentally exhausted and drained. (8) |  |  |  |  |  |

Mental distance

|  | Never (1) | Rarely (2) | Sometimes (3) | Often (4) | Always (5) |
| --- | --- | --- | --- | --- | --- |
| 9. I struggle to find any enthusiasm for my work. (1) |  |  |  |  |  |
| 10. At work, I do not think much about what I am doing and I function on autopilot. (2) |  |  |  |  |  |
| 11. I feel a strong aversion to my job. (3) |  |  |  |  |  |
| 12. I feel indifferent about my job. (4) |  |  |  |  |  |
| 13. I'm cynical about what my work means to others. (5) |  |  |  |  |  |

Cognitive impairment

|  | Never (1) | Rarely (2) | Sometimes (3) | Often (4) | Always (5) |
| --- | --- | --- | --- | --- | --- |
| 14. At work, I have trouble staying focused (1) |  |  |  |  |  |
| 15. At work, I struggle to think clearly (2) |  |  |  |  |  |
| 16. At work, I'm forgetful and distracted (3) |  |  |  |  |  |
| 17. At work, I have trouble concentrating (4) |  |  |  |  |  |
| 18. I make mistakes in my work because I have my mind on other things (5) |  |  |  |  |  |

Emotional impairment

|  | Never (1) | Rarely (2) | Sometimes (3) | Often (4) | Always (5) |
| --- | --- | --- | --- | --- | --- |
| 19. At work, I feel unable to control my emotions (1) |  |  |  |  |  |
| 20. I do not recognise myself in the way I react emotionally at work (2) |  |  |  |  |  |
| 21. During my work I become irritable when things don't go my way (3) |  |  |  |  |  |
| 22. I get upset or sad at work without knowing why (4) |  |  |  |  |  |
| 23. At work I may overreact unintentionally (5) |  |  |  |  |  |

Start of Block: Section 2B: Burnout Secondary Symptoms

Psychological complaints (general)

|  | Never (1) | Rarely (2) | Sometimes (3) | Often (4) | Always (5) |
| --- | --- | --- | --- | --- | --- |
| 1. I have trouble falling or staying asleep (1) |  |  |  |  |  |
| 2. I tend to worry (2) |  |  |  |  |  |
| 3. I feel tense and stressed (3) |  |  |  |  |  |
| 4. I feel anxious and/or suffer from panic attacks (4) |  |  |  |  |  |
| 5. Noise and crowds disturb me (5) |  |  |  |  |  |

Psychosomatic complaints (general)

|  | Never (1) | Rarely (2) | Sometimes (3) | Often (4) | Always (5) |
| --- | --- | --- | --- | --- | --- |
| 6. I suffer from palpitations or chest pain (1) |  |  |  |  |  |
| 7. I suffer from stomach and/or intestinal complaints (2) |  |  |  |  |  |
| 8. I suffer from headaches (3) |  |  |  |  |  |
| 9. I suffer from muscle pain, for example in the neck, shoulder or back (4) |  |  |  |  |  |
| 10. I get sick (5) |  |  |  |  |  |

Start of Block: Additional information

Is there anything else about your experience you would like to add? (Please note: there is also an option to participate in an interview, see more details upon completion of questionnaire)

- No (1)
- Yes: (2) ________________________________________________

End of Block: Additional information
